# Response to Hron *et al.*

**DOI:** 10.1186/s13059-015-0725-y

**Published:** 2015-08-18

**Authors:** Peter V. Lovell, Morgan Wirthlin, Lucia Carbone, Wesley C. Warren, Claudio V. Mello

**Affiliations:** Department of Behavioral Neuroscience, Oregon Health and Science University, Portland, OR USA; The Genome Institute, Washington University School of Medicine, St Louis, MO USA; Oregon National Primate Research Center, West Campus, Oregon Health and Science University, Portland, OR USA

**Keywords:** Gene loss, Genome assembly, Gene loss, Synteny verification

## Abstract

Hron *et al.* provide transcriptome evidence that three (1.1 %) of the 274 genes reported by Lovell *et al.* as missing in birds may actually be ‘hidden’ as a result of high GC content. Although this factor may explain some gene absences from genomic assemblies, we believe it is insufficient to account for the extensive syntenic losses described in Lovell *et al.*

Please see related article: www.dx.doi.org/10.1186/s13059-015-0724-z

We recently reported in Lovell *et al.* [1] 274 genes that are missing from the genomes of 60 avian species, but present in the genomes of other vertebrate lineages. We also reported 174 genes (Supplemental Tables S6A and B in [1]) that we were unable to find in chicken but that are present in other avian genomes (that is, that are not lost in birds). Hron *et al.* [2] now report that a small subset of these missing genes, particularly those we reported as missing in chicken only, can actually be found in chicken by mining “large amounts of ‘raw’ next-generation sequence data available from the Sequence Read Archive (SRA)”, derived from RNA-seq datasets. The authors [2] argue that these genes, together with others not examined by Lovell *et al.* [1], contain relatively long stretches of sequence with very high GC content, which probably made them more difficult to assemble, and thus resulted in their apparent absence from the chicken and (in some cases) other avian genomes. They go on to imply that several other genes reported by Lovell *et al.* [1] as missing “can be expected to be assembled from SRA data”. Although we acknowledge that the communication by Hron *et al.* [2] provides a significant contribution by highlighting a possible role for GC content and stretches in the incompleteness of avian genome assemblies, we believe that the conclusion that several other genes “can be expected” is largely unsupported. Indeed, the authors [2] themselves conclude that “the vast majority of the genes reported in Lovell *et al.* [1] are probably really missing in birds”.

We point out that Hron *et al.* [2] were able to find only three of the main set of 274 missing genes that we reported [1]. We consider this to be a very low and acceptable rate (about 1.1 %) of false positives (that is, mistakenly reported as missing), given the highly comprehensive scale of our efforts. Moreover, several other genes that Hron *et al.* [2] report as present in chicken are derived from a much lower confidence list of 89 genes (Supplemental Table S6A in Lovell *et al.* [1]) that we did not find in chicken but found in one or several other bird species based on RefSeq evidence. It is thus not too surprising that some of these have eventually also been found in chicken. We note that we found yet another set of 85 genes not found in chicken (Supplemental Table S6B in [1]) by conducting thorough searches of NCBI’s whole genome shotgun databases of 60 avian genomes; it would not surprise us if some or all of these are also eventually found in chicken. On the other hand, Hron *et al.* [2] do not clarify the full extent of their raw read searches, and do not comment on whether they also failed to uncover evidence for other missing genes. Without addressing this possibility, the statement that several more genes from the main set in [1] “can be expected” to be found in chicken seems unsupported. We also note that although we have verified that the sequences assembled by Hron *et al.* [2] do align to the presumed orthologs in multiple non-avian species, the authors do not clearly explain how the sequences were assembled, nor do they provide clear synteny evidence, leaving open the concern that some of these could represent paralogs or related gene family members. Notably, at least 12 of the lizard-human orthologs that are missing in birds actually have one (or more) closely related paralogs (Supplemental Table S3 in [1]). Thus, definitive conclusions about orthology may require further sequence and/or assembly data that provide context for the newly found sequences.

The main set of 274 missing genes we reported [1] represent genes that are preserved in syntenic blocks in non-avian organisms. It seems unlikely that all these syntenically organized genes have similarly high GC content and structure, and that this factor alone accounts for their being ‘hidden’ in avian genomes. That possibility, if true, would represent a remarkable new insight into genome organization. It seems more parsimonious to interpret their absence as a loss resulting from chromosomal rearrangements, for which we did provide supportive evidence (for example, Figure four and Figure S1 in [1]). We were also very cautious and conservative in not including in the main missing set about 110 genes for which only very fragmentary evidence was available and no synteny confirmation was possible (Supplementary Table S18 in [1]). This set matches closely Hron *et al.* [2]’s description as “absent from the current chicken assembly, or are present only as small fragments in unidentified genomic contigs”. We also find it noteworthy that a considerable set of high GC genes have been sequenced and assembled within a reasonable syntenic context in the Tibetan tit, even though, as Hron *et al.* [2] have found, their average GC content in the tit is not significantly different from that observed in other avian species. This indicates that high GC content is not an absolute impediment to sequencing, assembling and inclusion in current avian genome databases. It also raises the intriguing question of why these sequences are so well represented in this species, given that the same technology (Illumina) was used as for most other current avian genomes in NCBI where such genes cannot be found.

The report by Hron *et al.* [2] has considerable value in that it highlights the importance of GC content, but we believe that the evidence presented does not significantly alter the main findings reported in [1]. Given that genes frequently absent from the current databases have very high GC content and low overall conservation when compared with non-avian organisms, the most important emerging question for future studies is arguably whether such genes are transcriptionally active and have conserved the biological function of their non-avian orthologs, or whether they might be considered ‘functionally absent’ in birds.

## References

[1] Lovell PV, Wirthlin M, Wilhelm L, Minx P, Lazar NH, Carbone L (2014). Conserved syntenic clusters of protein coding genes are missing in birds. Genome Biol.

[2] Hron T, Pajer P, Paces J, Bartunek P, Elleder, D. Hidden genes in birds. Genome Biol. 2015; in press. 10.1186/s13059-015-0724-z PMC4539667 26283656

